# Protocols of Investigation of Neonatal Cholestasis—A Critical Appraisal

**DOI:** 10.3390/healthcare10102012

**Published:** 2022-10-12

**Authors:** Patricia Quelhas, Joana Jacinto, Carlos Cerski, Rui Oliveira, Jorge Oliveira, Elisa Carvalho, Jorge dos Santos

**Affiliations:** 1 Faculty of Health Sciences, Health Science Investigation Center of University of Beira Interior (CICS-UBI), 6200-506 Covilha, Portugal; 2 Medicine Department, University of Beira Interior (UBI), Faculty of Health Sciences, 6201-001 Covilha, Portugal; 3 Pathology Department of Universidade Federal do Rio Grande do Sul (UFRGS), Pathology Service of Hospital de Clínicas de Porto Alegre (HCPA), Porto Alegre 90035-903, Brazil; 4 Centro de Diagnóstico Histopatológico (CEDAP), 3000-377 Coimbra, Portugal; 5 Center for Predictive and Preventive Genetics (CGPP), IBMC, UnIGENe, i3S, University of Porto, 4200-135 Porto, Portugal; 6 Department of Gastroenterology and Hepatology, Hospital de Base do Distrito Federal, Hospital da Criança de Brasília, Brasília 70330-150, Brazil

**Keywords:** cholestasis, neonatal, diagnosis, differential, biliary atresia, cholestasis, neonatal intrahepatic

## Abstract

Neonatal cholestasis (NC) starts during the first three months of life and comprises extrahepatic and intrahepatic groups of diseases, some of which have high morbimortality rates if not timely identified and treated. Prolonged jaundice, clay-colored or acholic stools, and choluria in an infant indicate the urgent need to investigate the presence of NC, and thenceforth the differential diagnosis of extra- and intrahepatic causes of NC. The differential diagnosis of NC is a laborious process demanding the accurate exclusion of a wide range of diseases, through the skillful use and interpretation of several diagnostic tests. A wise integration of clinical-laboratory, histopathological, molecular, and genetic evaluations is imperative, employing extensive knowledge about each evaluated disease as well as the pitfalls of each diagnostic test. Here, we review the difficulties involved in correctly diagnosing the cause of cholestasis in an affected infant.

## 1. Definition

Cholestasis is defined as an anatomical or functional blockade to the biliary flow irrespective of the cause and site of obstruction, resulting in the accumulation of bile products in the liver, blood, and other tissues. Biochemically, cholestasis is characterized by increased levels of direct-reacting (conjugated) bilirubin, bile acids or their intermediate metabolites, and other bile compounds. From a histopathological point of view, there is an accumulation of bile pigment in hepatocytes and biliary canaliculi. Cholestasis starting in the first 3 months of life is named Neonatal cholestasis (NC) [1]. NC presents an incidence of 1:2500 live births [2,3]. Jaundice, hypocholic stools, and choluria are features of NC, although anicteric infants or presenting normal stools can present NC. Occasionally, steatorrhea or profuse bleeding can be the first signs of NC [4]. In infants, which present almost colorless urine, choluria does not mean “dark urine” as in cholestatic adults, but instead yellow-colored urine that stains diapers. Cholestasis is defined as a direct serum bilirubin level above 1 mg/dL at a total bilirubin value of up to 5 mg/dL or when the direct fraction is higher than 20% at total bilirubin over 5 mg/dL [5]. In clinical practice, direct bilirubin serum levels of 1 mg/dL or more are sufficiently accurate to indicate NC [6,7,8]. In the first 5 days of life, even direct or conjugated bilirubin serum levels as low as 0.3–0.4 mg/dl and 10% of the total bilirubin are suggestive of cholestasis [9].

## 2. Differential Diagnosis

Two etiological groups cause NC: extrahepatic obstructive and intrahepatic diseases (Table 1). The differentiation between these two groups is urgent since prompt therapeutic approaches for some specific diseases can prolong the native liver survival and be lifesaving. The most frequent cause of NC is Biliary atresia (BA), an infantile hepatobiliary disorder consisting of both extrahepatic bile duct obstruction and intrahepatic fibrosing cholangiopathy. In the presence of BA, surgical release of the extrahepatic blockage through a Portoenterostomy can defer the development of cirrhosis [10]. The postoperative prognosis of Portoenterostomy is affected by the age at the procedure [11,12]. Although the procedure performed until 45 days of life avoids the need for liver transplantation (LTx) up to 16 years old [13], even when portoenterostomy is timely performed under ideal conditions of care, cholangiopathy leads to the need for liver transplantation in 78% of the patients until the third decade of life [14]. A realistic goal of Portoenterostomy is not lifelong avoidance of LTx but rather to postpone its need as long as possible. Concerning intrahepatic disorders, available therapies instituted promptly can protect against the toxic effects of bile acids or their intermediate metabolites in the liver [15].

A decreasing percentage of cases of NC remains classified as “idiopathic neonatal hepatitis” [16], a term used to denote a clinicopathological syndrome with an undefined cause. The unveiling of the molecular basis of diverse subsets of NC has offered opportunities for expanding diagnostic tools and treatment strategies.

In the neonatal period, immaturity in the bile acid synthetic and metabolic pathways results in increased susceptibility of the liver to toxic insults. This neonatal propensity to cholestasis worsens with both low gestational age and birth weight, enhancing the chance of liver disease during infections, drug therapy, and intestinal failure. Intestinal failure precludes enteral nutrition and requires using parenteral formulas, increasing the risk of NC [4,17,18,19]. On the other hand, the occurrence of transient NC, that is, that disappears after six months, is increasing. In these cases of spontaneous disappearance of NC, it is crucial to rule out associated liver diseases presenting their prodromes [20].

## 3. The First Challenge: Pitfalls in the Diagnosis of Biliary Atresia

**Figure 1:**
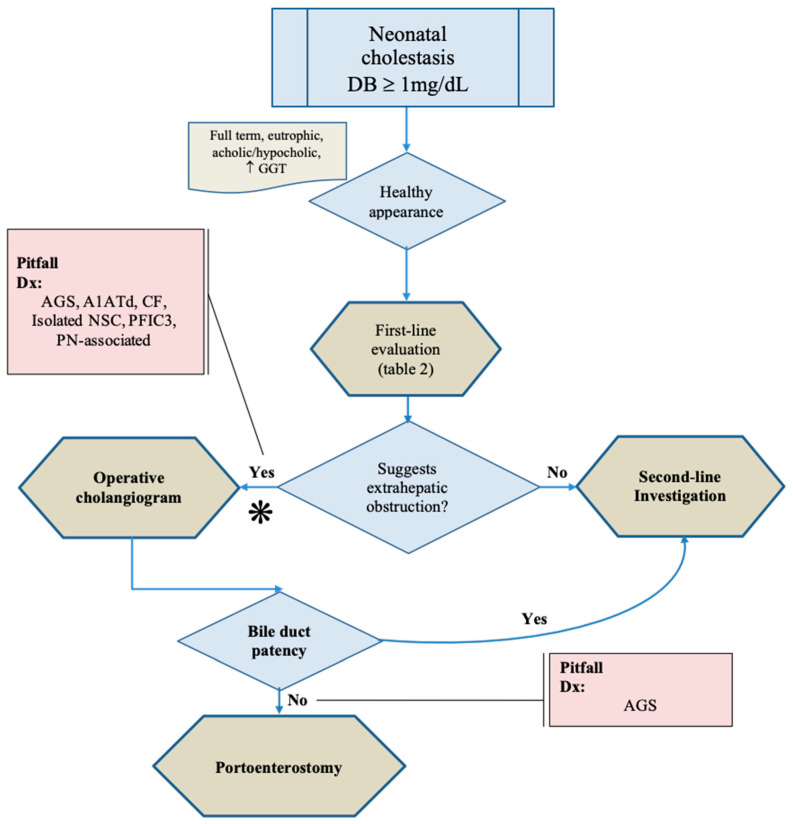
**Algorithm for differential investigation between Biliary atresia and intrahepatic diseases.** Healthy appearance in an infant with clinical-laboratory (including increased GGT serum levels), image, and histopathological findings suggestive of extrahepatic obstruction indicates the need to perform an operative cholangiogram, since some intrahepatic diseases can mimic biliary atresia. Even in the operative cholangiogram remains the risk of a false-positive diagnosis of biliary atresia due to the presence of biliary ductal hypoplasia, or another ductal obstructive lesion, associated with Alagille syndrome. * —increased MMP serum levels and presence of subcapsular telangiectasias with dilated vessels may be accurate diagnostic tests at this point (to be confirmed). Abbreviation: AGS—Alagille syndrome; Dx—differential diagnosis; NSC—neonatal sclerosing cholangitis; PN—parenteral nutrition. Source: authors illustration.

Differentiating in time NC caused by an extrahepatic obstruction from intrahepatic disorders is still challenging. Figure 1 presents a suggestion of an algorithm for the diagnostic differentiation between BA and intrahepatic causes of NC.

From a clinical perspective, NC in a thriving infant with a healthy appearance, increased GGT serum levels, and long-lasting (7 days or more) acholic stools, indicates the diagnosis of BA [23]. However, this should not be seen as an infallible rule (Table 2).

Among the methods used to differentiate between extra- and intrahepatic causes of NC, abdominal ultrasound is usually the first to be performed, since it is a non-invasive test, rules out other extrahepatic disorders, and identifies the biliary atretic process by the “triangular cord” sign [28]. However, liver hilum inflammation can hide the “triangular cord” sign, thus decreasing the method sensitivity [29]. Absence of gallbladder, abnormal gallbladder shape, enlargement of the hepatic arterial lumen, and subcapsular blood flow are findings that increase the ultrasound diagnostic accuracy for BA, but inter-observer disagreement can occur [30,31,32]. Ultrasound may not differentiate between BA and intrahepatic causes of cholestasis in infancy such as Alagille syndrome and other intrahepatic diseases [33,34,35]. The clinical and laboratory scores to distinguish between BA versus non-BA using laboratory data available at clinical presentation need to be confirmed through multicenter studies. The presence of firm hepatomegaly historically considered indicative of BA [36], although suggestive of BA, cannot be taken for a granted diagnosis of this disease. Inborn errors of metabolism involving the liver including alpha-1-antitrypsin deficiency (A1ATd) and cystic fibrosis can lead to firm hepatomegaly [37,38]. In addition, GGT serum levels which are greatly increased in BA [23], are often very high also in Alagille syndrome, sclerosing cholangitis, and in any disorder with extensive biliary structural involvement [23,24,39].

**Figure 2:**
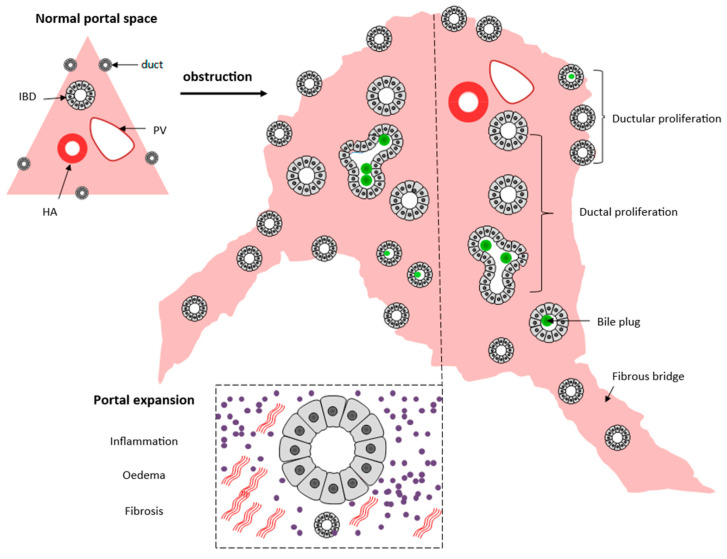
**Schematic presentation of the histopathological extrahepatic obstructive pattern neonatal cholestasis**. Upper left: portal tract with the portal triad, and bile ductules lining the portal/parenchymal interface. In biliary obstructive disorders, there is a portal expansion caused by ductular reaction, inflammatory cell infiltration, edema, and fibrosis. Proliferated ducts and ductules present bile plugs in the lumen. Fibrosis and ductular reaction surpass the limits of the portal tract forming fibrous (portal-portal and/or portal-venous) bridges. Source: authors illustration.

The analysis of a percutaneous liver biopsy is a valuable diagnostic tool in the initial evaluation of a cholestatic infant, presenting high levels of accuracy [40,41,42]. It depends on the finding of an obstructive pattern, which is depicted in Figure 2.

**Figure 3:**
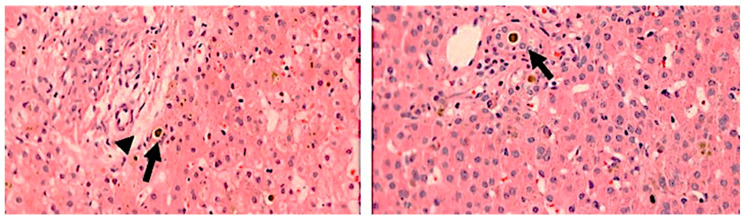
**Dynamic histopathological features in the liver of a patient with biliary atresia.** A—21-day-old patient with biliary atresia (preoperative percutaneous biopsy): absence of ductular reaction and presence of canalicular cholestasis (arrow). Arrowhead: portal tract limiting plate. B—Portal space from the same patient 30-day old, in a wedge liver biopsy obtained at the exploratory laparotomy. There is ductular reaction with the presence of bile plugs in neoductules (arrow). Hematoxylin-eosin, lens: 20×. Source: image obtained at the Department of Pathology, Hospital de Clínicas de Porto Alegre, Brazil, and published under permission of such Service.

However, the obstructive characteristics develop in a time-dependent manner (Figure 3) and also occur in Alagille syndrome, A1ATd, cystic fibrosis, parenteral nutrition-associated NC (PNALD), PFIC3, and isolated neonatal sclerosing cholangitis (gene DCDC2) [43]. In addition, typical histologic features for intrahepatic disorders quoted in the literature often are not observed in the realm of this urgent diagnostic challenge. For instance, the bile duct paucity characteristic of Alagille syndrome requires documentation of a ratio of interlobular bile duct/hepatic artery branch in the portal tracts lower than 0.4 [44]. The ideal number for this task is 20 portal tracts, which is unachievable in percutaneous biopsies. As many as five or more portal tracts are acceptable but less accurate. The ratio of bile duct/hepatic artery varies according to the stage of biliary system development and changes even beyond birth. Finally, the references used for adults and infants may differ, especially for premature babies, leading to an overdiagnosis of bile duct paucity in this age group [42,44]. Bile duct paucity may arise not only as a developmental defect but may also be secondary to ischemia, atrophy, toxicity, and inflammation.

**Figure 4:**
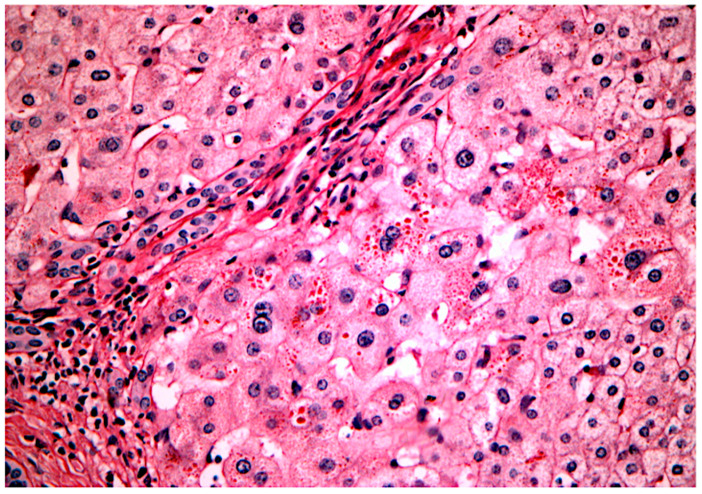
**Alpha-1 antitrypsin globules inside periportal hepatocytes in a patient with A1AT-associated liver disease**. Periodic acid–Schiff stain (PAS) with diastase clearing, lens: 40×. Source: image obtained at the Department of Pathology, Hospital de Clínicas de Porto Alegre, Brazil, and published under permission of such Service.

On the other hand, the presence of diastase-resistant PAS-positive globules is strongly suggestive of A1ATd (Figure 4) but these structures only appear after the 12th week of life [45].

Intraoperative cholangiography is considered the gold standard for diagnosing BA, but the method can be misleading in up to 20% of cases [7,46,47,48,49]. Pathophysiological considerations can help us understand why diagnostic errors occur in intraoperative cholangiography. Extrahepatic bile flow blockage does not occur exclusively from complete mechanical obstruction caused by BA but also from extrahepatic bile duct hypoplasia caused by decreased intrahepatic bile flow, or extrahepatic bile duct agenesis both associated with Alagille syndrome [49,50,51]. Inadvertently submitting an infant with Alagille syndrome to a Portoenterostomy based on a false-positive diagnosis of BA can lead to grievous prognostic consequences [52,53]. Technical optimization of cholangiography by radiologists and pediatric surgeons, be it intraoperative or laparoscopic, can increase the method’s accuracy, and avoid an unnecessary portoenterostomy [29,54,55,56].

Given the pitfalls in the differential diagnosis of BA, a relevant trans-operative procedure for a final confirmation is the porta hepatis excision to enable the analysis of the biliary remnants by an experienced pathologist. The diagnostic confirmation may have prognostic implications for a specific patient with Alagille syndrome inadvertently submitted to a portoenterostomy; may serve as a tool for quality control evaluation of the clinical and surgical services, and for improving the accuracy of investigation results when the correct diagnosis of BA is an independent variable.

### 3.1. Novel Approaches for Identification of BA

Promising methods for accurately identifying BA have been recently proposed based on knowledge generated by basic sciences and imaging studies.

#### 3.1.1. Serum Markers

Potential markers of BA, such as cytokines linked to the pathogenesis of BA and other cholangiopathies, are under investigation [57,58]. Proteomic analysis of serum samples at the time of diagnosis of BA uncovered high circulating levels of matrix metalloproteinase-7 (MMP-7) compared with normal and cholestatic controls. MMP-7, which is secreted by the normal epithelium and shows increased serum levels upon biliary injury, modulates the clinical phenotype in the experimental model of BA. The assessment of the serum levels of MMP-7 seems to be an accurate method for diagnosing BA, but large-scale populational studies of this non-invasive approach are still warranted [25,57,58,59,60,61].

#### 3.1.2. Arterial Vascular Abnormalities

**Figure 5:**
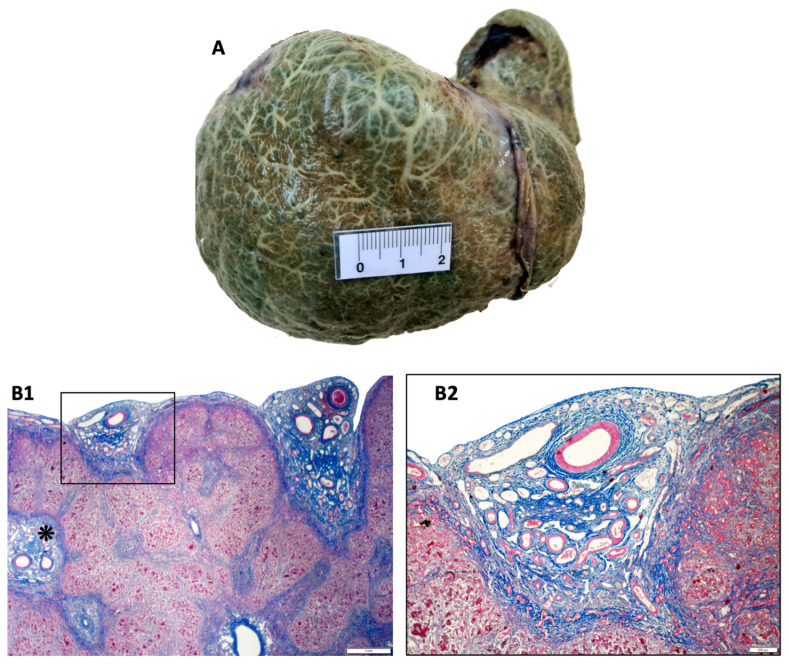
**Subcapsular telangiectasias in the explant of a patient with biliary atresia**—Macro anatomic image (**A**): diffuse extensive subcapsular telangiectasias in the liver explant. Microanatomic images: (**B1**) subcapsular vascular agglomerates. Asterisk—continuation of the same vascular features to a portal tract through a fibrovascular septum (lens: 10×); (**B2**) detail of the subcapsular vascular agglomerate presented in figure (**B1**): note the hyperplasia of arterial/arteriolar vessels, veins, and lymphatics within the subcapsular fibrous stroma. Observe the large ductular reaction at the interface with parenchyma. Source: image obtained at the Department of Pathology, Hospital de Clínicas de Porto Alegre, Brazil, and published under permission of such Service.

A novel area of investigation in the BA diagnosis comes from microanatomic studies on arterial vascular alterations observed in this disease. In the liver of BA patients, there is a proliferative pattern of arterioles and arteries, presenting prominent muscle layers, in portal tracts, fibrous septa, and the subcapsular region [62]. This type of altered arterial vascular pattern was also clearly evidenced by an arteriographic study, which showed specifically in BA patients at the time of portoenterostomy, peripheral arterial blockage with perivascular arterial tufts [63]. Eventually, several groups of investigators have observed image findings of increased luminal diameter of the hepatic artery at the liver hilum, and subcapsular vascularization specifically in BA patients, thus serving for the differential diagnosis between BA and cases of intrahepatic cholestasis [31,64,65,66,67,68,69,70]. Recently, laparoscopic studies performed before portoenterostomy confirmed that the observed subcapsular vascularization in BA is caused by spider telangiectasias with arterial vessels showing luminal dilatation. High accuracy for the diagnosis of BA was obtained with this method [26,71]. Figure 5 shows subcapsular telangiectasis in the explant of a patient with BA. These findings, however, need to be reproduced by other groups, and the mechanism of this arteriolar proliferation and hyperplasia should be investigated.

#### 3.1.3. Are Genetic Studies Useful to Differentiate BA from Intrahepatic Causes of NC?

Under the current diagnostic routines, the risk of a faulty diagnosis of BA remains, implying an unnecessary portoenterostomy. The intrahepatic diseases confusable with BA can often be distinguished but not always. And not rarely. Sometimes, laboratory tests are the gold-standard method, such as the sweat electrolytes and/or the determination of fecal elastase or immunoreactive trypsin for cystic fibrosis [27]. Concerning A1ATd, serum levels of α1AT can be assessed, and if decreased the deficient variant of the A1AT protein can be identified by protease inhibitor (PI) typing through polyacrylamide isoelectric focusing (PI-M, PI-S, PI-Z alleles) [27,72]. However, A1AT is an acute phase reactant and misleading results of the A1AT serum levels occur in the presence of systemic inflammation [7]. Alagille syndrome can present with syndromic features, but sometimes these characteristics are absent at the time of the NC investigation, making its identification difficult with the routinely used methods. Isolated neonatal sclerosing cholangitis is a rare disease that can be confused with BA, and although PFIC3 tends to start later in childhood, it also deserves consideration.

A first approach to exclude some of these confusable diseases is to include tests that diagnose them in the first line of NC evaluation (Table 2, Echocardiogram and Others). Could single-gene analysis for *JAG1*, *NOTCH2* (Alagille syndrome), *SERPINA 1*(A1ATd), *DCDC2* (isolated neonatal sclerosing cholangitis), and *ABCB4* (PFIC3) still be used to exclude these intrahepatic diseases and avoid diagnostic errors in the first step of the investigation? The first difficulty in using single-gene tests for this aim is the turnaround time presently needed to obtain the results. Concerning only a single-gene test for monogenic diseases, experienced centers can have data adequately analyzed after ten days at least. However, in the investigation of NC worldwide, TGS results become available after thirty or even ninety days. That is too long for the first step of NC investigation, as for decisions involving NC associated with fulminant liver failure. Another limitation is around the possibility of identifying variants of unknown clinical significance (VUS) is genetic studies. Multigene analysis would allow simultaneous analysis of several genes and may circumvent the limitation of facing one VUS in one gene, but the actual disease-causing variant maybe present in another locus.

Presently, the decision to perform a portoenterostomy cannot depend on genetic analysis since pathogenic variants, even those associated with Alagille syndrome or A1ATd, occur in patients with BA. In the case of A1ATd, the observed pathogenic variant of the *SERPINA1* gene can be the true cause of NC or can be a coincidental finding that may be acting as an influencing gene [73,74]. In a small study, we retrospectively evaluated 28 patients with NC treated in the Pediatric Hospital of Centro Hospitalar e Universitário de Coimbra (CHUC) through a targeted gene panel including 54 genes related to NC and performed in CGPP Laboratory (IBMC, i3S, UP). Relevant genetic variants were identified in 19/28 (68%) patients with NC. Among 15 patients diagnosed as BA, 80% presented relevant genetic variants, and 3 of these BA patients showed a molecular diagnosis suggestive of intrahepatic diseases, including A1ATd (*n* = 1), Cystic fibrosis (*n* = 1) and AGS (*n* = 1). Given the retrospective nature of the study, we could not ascertain if these pathogenic variants were coincidental findings between BA and intrahepatic diseases, maybe with effects over clinical severity [75] or false-positive diagnosis of BA [76]. These findings reinforce the need for confirmation by a pathologist of the diagnosis of BA through the examination of the biliary remnants within the excised porta hepatis.

Additional pitfalls come from the technical difficulties inherent to next-generation sequencing (NGS) performance, and variant interpretation as discussed below.

## 4. The Second Challenge: The Identification of the Neonatal Intrahepatic Disease

After excluding surgically correctable extra-hepatic obstruction, the next step is the differentiation of intrahepatic disorders whether they are currently treatable or not. For treatable intrahepatic causes of NC including infection, galactosemia, tyrosinemia type 1, hereditary fructose intolerance, hypothyroidism, cystic fibrosis, hypopituitarism, and bile acid synthesis defects timely therapeutics can be life-saving or at least reduce the noxious effects of the infectious or metabolic derangement [7,77,78,79]. Around 25–50% of cases of NC represent monogenic disorders with autosomal recessive inheritance caused by homozygous or double heterozygous variants (See Supplemental File, Figure S1) except for Alagille syndrome [78].

The use of NGS brought a new era in this step of NC evaluation [9,77], constituting a novel paradigm for the attainment of diagnosis and treatment choice [78,79]. In children with suspected genetic diseases, the diagnostic and clinical utility of NGS shows better results than chromosomal microarray study, especially when resorting to trios (patient and both parents) [80]. However, the mistime use of genetic testing or without adequate patient selection can lead to uninterpretable information, complicating the diagnosis. The clinical-laboratory investigation of the patient with NC should serve as the basis for the differential investigation, including the NGS method information. Clinical suspicion should direct the investigation in terms of NGS multigene panels, either based on whole-exome sequencing (WES) or custom capture of genes of interest, but always in association with confirmatory molecular, histopathological, and imaging analyses, as necessary. Presently, in many centers turnaround time for obtaining NGS results may exceed what is desirable in terms of agility and accuracy for the best possible treatment. In some contexts, there are technical limitations intrinsic to the NGS method more currently used, such as a decreased sensitivity due to incomplete capture of target regions and existence of high homology regions (such as for the case of pseudogenes), raising the possibility of causal variants being not identified by the genetic screen. An important challenge is the accurate integration of the genetic data with the true clinical picture through bioinformatic analysis and this difficulty increases with the number of genes under evaluation [81]. The prediction of variant pathogenicity is challenging in the clinical setting. A VUS represents a suspended diagnosis in clinical situations that may require precise and rapid management. In the case of highly suspicious variants, deepening the disease evaluation with clinical reevaluation, biochemical, image, histopathological, transcriptome, and proteomic analyses may become mandatory. In fact, around 30% of patients presenting PFIC-like features have no identified disease-causing variants of the known genes associated with PFICs [82]. Moreover, the existence of copy number variations, structural rearrangements such as translocation or inversion, partial gene rearrangements, and even variants in the promoter or intronic regions which have important effects on cellular function, add technical complexity to an accurate diagnosis. Moreover, phenotypic variability among patients with the same pathogenic variant is a recognized interpretation difficulty. The role of heterozygous pathogenic variants in the development of NC gives rise to additional difficulties in the correct interpretation of NGS [83].

In the evaluation of a suspect case of Alagille syndrome, an autosomal dominant genetic disorder with variable penetrance and clinical expression, obtaining a non-diagnostic NGS result implies the need for additional genetic tests to identify structural rearrangements such as copy number variations of DNA regions [78].

### 4.1. Clinical-Laboratory Investigation

#### Acutely-Ill Appearing Child

**Figure 6:**
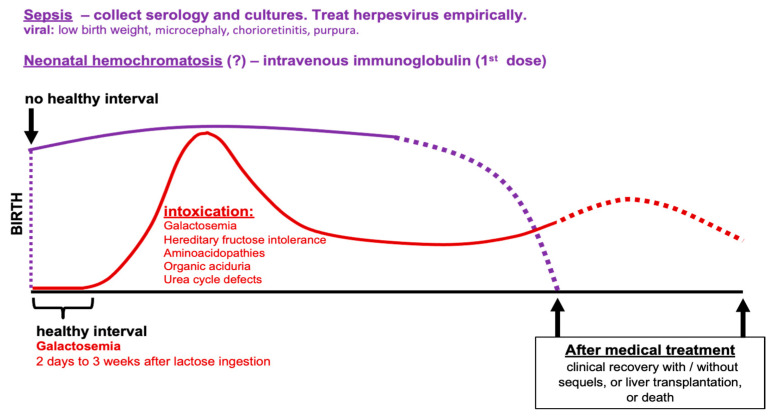
**Comparison of initial clinical behavior of metabolic intoxications and other severe neonatal liver diseases**. Source: authors illustration based on concepts from [88].

Clinical-laboratory investigation is the primary approach for treating the emergency of an ill-appearing infant with NC. Many acutely ill-appearing infants with NC suffer from treatable disorders and timely diagnosis is a crucial task to avoid acute liver failure or avoid a disease chronification. Acute liver failure is diagnosed in the presence of a marked increase in direct bilirubin serum levels, fast increasing aminotransferases, coagulopathy unresponsive to vitamin K (INR ≥ 1.5, or even without encephalopathy if INR ≥ 2), in addition to hypoglycemia and increased serum ammonia [84]. From 28 days of life on, encephalopathy can be suspected if irritability, crying spells, and alterations in the sleep rhythm are evident [85]. Ill-appearance in a cholestatic infant is an emergency, caused by several disorders, many of them included in the group of inborn errors of metabolism (IEM). IEM are mostly inherited in an autosomal recessive manner and result from deficient activity of a single enzyme in a metabolic pathway. They are usually a differential diagnosis of sepsis, encephalopathy, or persisting common conditions such as infection. NC is a common manifestation of IEM with or without renal involvement [37]. Bacterial or viral sepsis are important differential diagnoses of IEM. In the case of infection, the clinical picture commonly starts at birth, while in IEM characterized by metabolic intoxication, such as galactosemia, hereditary fructose intolerance, disorders of amino acid metabolism, organic acidurias, and urea cycle defects, there is a healthy interval from the time of exposure to the insulting agent until beginning the clinical picture (Figure 6) [85]. In galactosemia, the healthy interval can be as short as 48 h or as long as 3 weeks. Newborn screening (NBS) tests for IEM can be lifesaving: a baby who looks ill and presents an NBS with elevated total galactose serum levels must be immediately investigated and treated as classic galactosemia [86] (Supplementary Material, Table S1a). On the other hand, in an ill-looking infant at birth presenting additional clinical signs such as low birth weight, microcephaly, chorioretinitis, or purpura, a viral infection must be strongly suspected. Serology and cultures (Table 2) must be collected immediately, but if herpes virus infection, an early fatal and curable disease, is suspected, empirical treatment must be initiated even before test results. Syphilis, rubella, and toxoplasmosis are additional agents presenting in the first day of life and leading to NC with associated coagulopathy and growth restriction [7].

In the classic genotype of galactosemia (including Q188R/Q188R variant of the *GALT* gene), there are absent or markedly reduced erythrocyte Galactose-1-phosphate uridyl transferase (GALT) enzyme activity, markedly elevated blood galactose and erythrocyte galactose-1-phosphate levels, and the patient is at risk to develop potentially lethal E. coli sepsis, as well as the long-term diet-independent complications of galactosemia. Recurrent *E. coli* sepsis in a neonate suggests galactosemia. Galactsosemia caused by *GALT* variant S135L/S135L may also lead to acute disease in the neonatal period including liver disease, growth failure, and cataracts [85,87,88].

NBS positivity for galactosemia does not necessarily imply classic galactosemia, but if an infant looks ill in the first days or weeks of life, classic galactosemia must be suspected even without markedly elevated total galactose level. In classic galactosemia there are poor feeding, vomiting, hypoglycemia, diarrhea, lethargy/coma, hypotonia, bulging anterior fontanel, hepatomegaly, jaundice (both direct-reacting and indirect bilirubin increases), bleeding diathesis, metabolic acidosis, Gram-negative (Escherichia coli) sepsis, and encephalopathy, with a high risk of death if untreated. Magnetic resonance of the brain shows alterations caused by cytotoxic edema and neuronal galactitol accumulation. Laboratory shows markedly decreased or undetectable GALT activity in red blood cells, and increased levels of plasma galactose, erythrocyte galactose-1-phosphate, and plasma and urine galactitol. Technical details in the biological sample collection are crucial: plasma and serum must be collected before any blood transfusion and immediately ultra-frozen stored [85].

Tyrosinemia type 1 is an autosomal recessive disorder characterized by a lack of activity of the fumarylacetoacetate hydrolase enzyme (FAH), leading to the accumulation of blood tyrosine, succinyl acetoacetate, and succinyl-acetone. In infants, it can present as an NC associated with acute liver failure, kidney tubular dysfunction, hypophosphatemic rickets, failure to thrive, and neurologic crises thus accounting for elevated early mortality. Later in childhood, it can manifest as cirrhosis or hepatocellular carcinoma. NBS for tyrosinemia type 1 can be lifesaving [89,90]. The diagnosis of tyrosinemia in infants is suggested by NC as associated with impending or overt acute liver failure and increased α-fetoprotein serum levels in the face of only mildly elevated aminotransferases. Renal dysfunction leads to glycosuria, phosphaturia, proteinuria, and aminoaciduria [91,92]. A small number of infants, mostly the premature and receiving high protein diet, may present transient tyrosinemia in the first 2 weeks of life, showing increased plasma tyrosine levels, lethargy, poor feeding, and decreased motor activity, although most are asymptomatic and identified through NBS [93].

Hereditary fructose intolerance is caused by a deficiency of the enzyme aldolase B (gene *ALDOB*) resulting in the accumulation of the toxic metabolite fructose-1-phosphate and in depletion of phosphate molecules indispensable for restituting the hepatic ATP [94,95]. Affected infants who ingest fructose develop NC associated with a severe and acute clinical picture, including nausea, vomiting, abdominal pain and distension, ascites, and hepatomegaly. Laboratory findings include hypoglycemia, lactic acidemia, hypophosphatemia, hyperuricemia, hypermagnesemia, and hyperalaninemia. Eventually, patients will develop growth restriction and failure to thrive. Fructose is in most oral medications, including vaccines and many formulas. Pacifiers are sometimes sugar dipped to soothe infants. Implementation of complete dietary restriction of the offending molecules early in life with maintained adherence can result in a good prognosis while, otherwise, liver and renal impairment ensue [94,95].

Gestational alloimmune liver disease (GALD) is presently recognized as the cause of almost every case of neonatal hemochromatosis and represents a major cause of acute liver failure. Neonatal hemochromatosis is the clinical condition in which severe liver disease in the neonatal period is accompanied by extrahepatic siderosis similar to hereditary hemochromatosis. GALD is the causal process of fetal liver injury [96,97]. In GALD, IgG antibodies from a mother sensitized to fetal-derived antigens are directed specifically against fetal hepatocytes, unleashing innate immune response. Infants present, in addition to marked hyperbilirubinemia including both conjugated and non-conjugated portions, hypoglycemia, coagulopathy, hypoalbuminemia, and edema. Renal impairment and oliguria may occur. Laboratory evaluation shows a small increase in aminotransferases, very high serum levels of α-fetoprotein, high ferritin levels, low transferrin levels, and high iron saturation. Treatment with a combination of double-volume exchange transfusion to remove existing reactive antibodies followed immediately by administration of high-dose intravenous immunoglobulin to block antibody-induced complement activation leads to high survival rates without liver transplantation [98]. The use of one dose of intravenous immunoglobulin for any infant in liver failure is recommended whether neonatal hemochromatosis is being considered (Figure 6). Diagnosis can be confirmed through buccal mucosal (minor salivary gland), liver, skin, and/or muscle biopsy for detection of iron deposition, as well as magnetic resonance in other organs with the same purpose [98,99]. If neonatal hemochromatosis is confirmed an exchange transfusion shall be performed followed by administration of a second dose of intravenous immunoglobulin [98,100]. Given the high recurrence risk of neonatal hemochromatosis in the next pregnancy, with an elevated frequency of concept death, the preventive use of intravenous immunoglobulin to the mother is warranted [101].

### 4.2. Integrative Approach of Clinical-Laboratory, Molecular, Histopathological, and Genetic Investigation for the Diagnosis of NC

**Figure 7:**
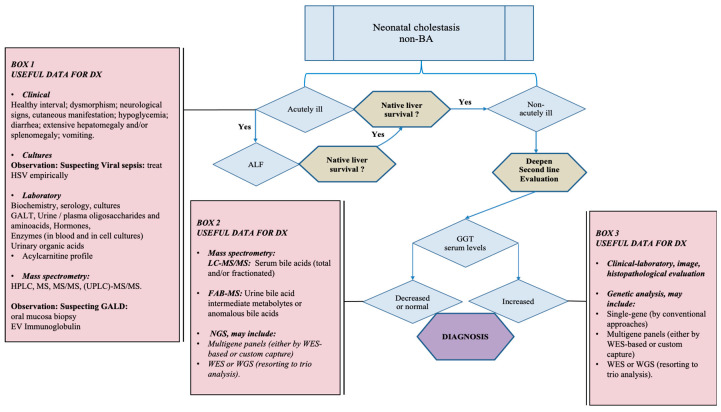
**Algorithm for differential diagnosis of neonatal intrahepatic cholestatic diseases.** Acute liver failure (ALF) must be discarded in a cholestatic infant presenting acutely ill signs. The urgent evaluation may include clinical examination, cultures, laboratory, molecular (including mass spectrometry) tests, and an oral biopsy (BOX 1) to make the differential diagnosis and permit a timely treatment. If the patient recovers, the second-line investigation can proceed. In the case of normal or decreased GGT serum levels, the bile acid contents in plasma and urine by mass spectrometry associated with NGS tests shall be made (BOX 2). Given that GGT serum levels are increased (BOX 3), clinical-laboratory, molecular, histopathological, and genetic investigation for the differential diagnosis. Concerning NGS, in situations of complex disorders, it is indicated to use Whole-exome sequencing and trio analysis instead of single-gene analysis (BOX 2 and 3). **Abbreviations not described in the text:** ALF—acute liver failure; Dx—differential diagnosis; EV Ig—intravenous immunoglobulin; FAB-MS-Fast Atom Bombardment Mass Spectroscopy; HSV—herpes simplex virus; HPLC—High performance liquid chromatography; LC-MS/MS; Liquid Chromatography—Tandem Mass Spectrometry (LC-MS-MS) MS—Mass spectrometry; MS/MS—Tandem Mass Spectrometry; TGS—targeted gene sequencing; UPLC—Ultra Performance Liquid Chromatography; WES—whole exome sequencing; WGS—whole genome sequencing. Source: authors illustration.

Figure 7 presents a suggestion of algorithm for the investigation of NC after excluding BA and other extrahepatic causes.

Reference centers of Pediatric Hepatology worldwide have diverse experiences in the investigation of NC often supported by Services of Clinical Genetics and Pediatric Surgery, equipped with specialized laboratories for complex biochemical enzymatic tests, and well prepared for invasive procedures such as percutaneous liver biopsy which is fairly safe in infants [102,103]. The clinical investigation paradigm in which “hypothesis rise from clinical and biochemical data and lead to genetic confirmation” [78] should not be replaced, but complemented by that in which the diagnostic algorithm starts from genetic screening. This is true at least presently given the pitfalls inherent to NGS and the complexities of NC [79]. The use of NGS is not imperative, or even feasible, in situations such as the first approach to metabolic intoxications, characterized by acute liver failure and whose diagnosis can be adequately performed with metabolic laboratory tests. In the case of galactosemia, however, TGS is a valuable option in the differential diagnosis between “Clinical Variant galactosemia” and classic galactosemia, with prognostic implications [88]. Concerning A1ATd, one of the most frequent causes of NC, diagnosis is efficiently performed through the assessment of α1AT serum levels, which if decreased, indicates the use of protease inhibitor (PI) typing through polyacrylamide isoelectric focusing or genotyping (PI-M, PI-S, PI-Z alleles) [27,72,78]. Cystic fibrosis, although not a frequent cause of NC, give rise to important prognostic implications for affected patients due to its life-threatening consequences [104,105,106,107]. The gold-standard test of Cystic fibrosis is an assessment of sweat electrolytes correctly performed [105] which can be complemented by fecal elastase, immunoreactive trypsinogen, and fecal fat excretion measurements for the evaluation of pancreatic function.

Table S1.h is based in Nicastro E and D’Antiga L (2018) and Nicastro E, et al. (2019). NGS is certainly the primary screening test for the investigation of NC associated with normal or decreased GGT serum levels. In this context, patients with increased bile acid concentration in plasma must be evaluated through single-gene analysis for PFICs, transaldolase deficiency, and familial hypercholanemia. In the case of patients with low GGT and reduced bile acid concentration in plasma, single-gene analysis is useful in the identification of specific defects of primary bile acid synthesis (Table S1.h.1). However, it is recommended a confirmatory assessment of bile acid intermediates and anomalous bile acids in urine through mass spectrometry [78,79]. Table S1.h.2 presents a suggested panel of diseases leading to NC with increased serum GGT levels. In this group of diseases, the NGS method has an important role in identifying the responsible pathogenic variants.

Some of the genetic diseases presented in Table S1.h are complex clinical disorders with compound phenotypes, involving the need for multigene panels, more specifically clinical exome, or WES, or even WGS, in trio evaluation, and thus demand intimate cooperation between geneticist, bioinformatic, and clinical teams with high expertise in the genetic investigation of NC [78]. In addition to NGS, it is crucial to integrate clinical-laboratory, molecular, and, when indicated, histopathological findings. For instance, in the investigation of lysosomal storage disorders commonly suspected due to the presence of large splenomegaly, the enzymatic tests can be performed in many centers by well-prepared specialized laboratories, and histopathology can be helpful. NGS studies present the advantage to identify the genetic basis of causal diseases, and from a prospective point of view, lead to the development of novel gene-therapeutic approaches [106]. On the other hand, the presence of NC associated with an acute severe clinical picture at birth or in the first days of life, with features of acute liver failure (Table S1.a), constitutes a challenge in any investigation algorithm and many patients are transplanted without a diagnosis. As previously discussed, an adequate clinical investigation followed by laboratory tests performed on an emergency basis can lead to diagnosis and offer adequate treatment for a reasonable proportion of patients. Extensive information useful for clinical-laboratory investigation can be found in Götze T, et al. (2015). Table S1 includes further useful references [107,108,109,110,111,112,113,114,115] concerning the clinical investigation. The development of projects for decreasing the turnaround time of TGS to less than 7 days in the situation of ill-appearing babies with NC under intensive care unit could optimize diagnosis, treatment, and prognosis.

### 4.3. The Role of Histopathological Investigation in the Differentiation of Intrahepatic Neonatal Cholestasis

All the diagnostic tests used in the investigation of NC present specific pitfalls involving intrinsic difficulties in carrying out the methods, the correct interpretation, and overlapping findings between different groups of diseases [116]. Presently, no preoperative isolated diagnostic test can with certainty identify BA, not even a cholangiogram, and in the first step of NC investigation, liver biopsy is yet useful, accurate enough [29,40,42,117] and, although invasive, a safe procedure in infants particularly when sonography-guided [7,102,103,118]. Liver biopsy goes beyond confirming after the neonatal period some expected diagnoses, such as Alagille syndrome or A1ATd, but can also reveal unexpected findings that can guide further diagnostic investigation, such as the possibility of metabolic liver diseases through the finding of microvesicular steatosis. Immunohistochemistry, immunolocalization of specific markers, and biochemical and molecular assays expand the information available through liver biopsy [119]. Histological evaluation and associated image analysis of the liver can help predict postoperative results after portoenterostomy [120] and define clinical prognosis in diseases such as NP-associated liver disorder [121,122,123]. Table S2 presents histopathological findings associated with intrahepatic NC.

Hepatitic findings are unspecific and result from the accumulation of cholephilic compounds in hepatocytes and Kupffer cells [116] with associated inflammation [124]. In addition to idiopathic NC, A1ATd, and BA, they occur at the initial presentation in several metabolic and hormonal diseases [125,126,127,128] and thus clinical correlation is mandatory. For instance, extensive giant cell transformation in a patient with normal GGT serum levels suggests PFIC2 or defects of bile acid synthesis [129,130,131] while the association of the findings organomegaly, ascites, and parental consanguinity increases incidence of autosomal recessive disorders, such as Niemann-Pick type C [132]. In this case, TGS can solve the diagnostic doubt. When clinical features of Zellweger spectrum disorders are suspected, an electron microscopy liver study is useful to reveal absent peroxisomes and anomalous mitochondria [133].

**Figure 8:**
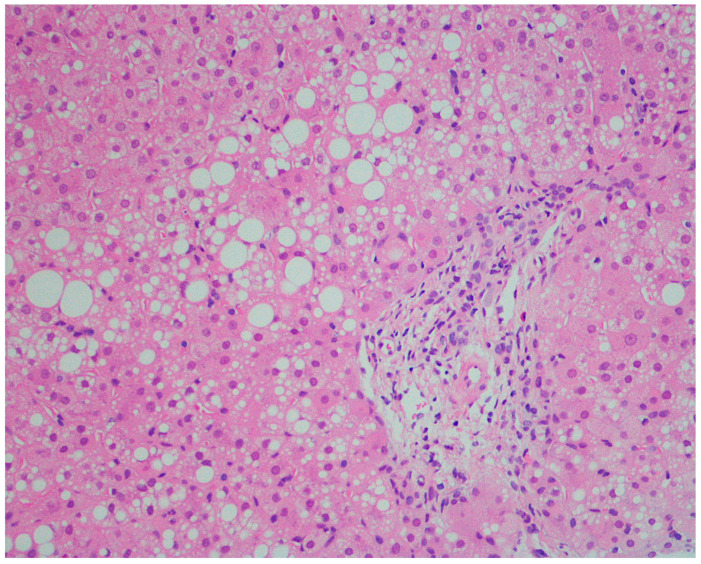
**Mixed steatotic pattern.** Hematoxylin-eosin, lens: 200×. Obtained at the Anatomopathology Service of the Hospital and University Center of Coimbra, and published under permission of such Service.

**Figure 9:**
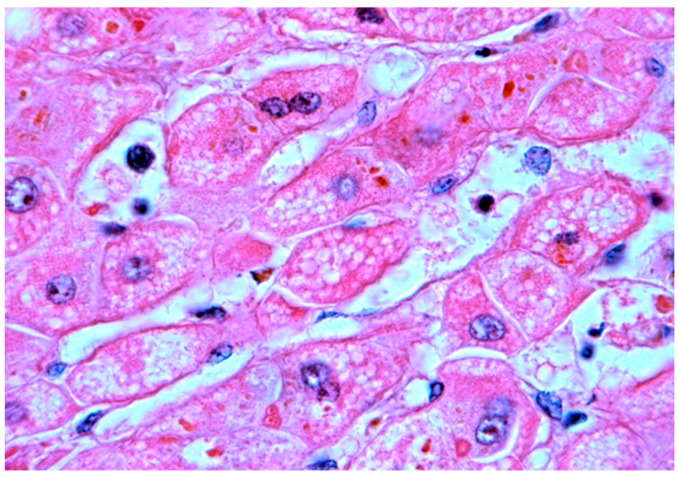
**Microvesicular steatosis in an infant with mitochondrial respiratory chain disorder.** Hematoxylin-eosin, lens: 100×, using oil immersion. Obtained at the Department of Pathology, Hospital de Clínicas de Porto Alegre, Brazil, and published under permission of such Service.

The steatotic pattern (Figure 8 and Figure 9) results from the hepatocellular accumulation of lipids attributable to increased fatty acid delivery, decreased hepatic fatty acid oxidation, impaired lipoprotein metabolism, and lysosomal storage [134].

Steatosis may present a macrovesicular, microvesicular, or mixed pattern. Macrovesicular cytoplasmic vacuoles displace the nucleus to the periphery, while in microvesicular steatosis nucleus remains at a central position. The form of steatosis more often found in infantile metabolic liver disease is macrovesicular and the occurrence of a microvesicular or a mixed pattern indicates the presence of diseases involving a mitochondrial pathology [125].

A frequent cause of the histopathologic steatotic pattern is Parenteral nutrition (PN)-associated NC. PN-associated NC occurs mostly in premature babies who cannot tolerate oral or enteral feedings and is related to high rates of early mortality. The hepatic complications of Total PN range from little increases in serum liver enzymes to steatosis, steatohepatitis, cholestasis, cholangitis, fibrosis, and cirrhosis [130]. Some hepatic lesions induced by Total PN are reversible, but persistent cholestasis with early cirrhosis can occur [135,136]. From a histopathologic perspective, at the time of an early diagnosis, there are light or moderate unspecific signs of NC, but in some patients, portal inflammation and necrosis are already present. The continuing use of PN gives rise to steatosis, steatohepatitis with intense cholestasis associated with a ductular reaction, portal inflammation, and progressive fibrosis. The detection of cirrhosis and of maintained elevated levels of serum bilirubin are both associated with an increased risk of death in the next 6-month period [121,122,123].

Metabolic intoxications also present the steatotic pattern but, given the association with acute liver failure, liver biopsy is often contraindicated in these disorders, and the diagnosis relies on the previously reported laboratory and/or genetic tests. Occasionally a liver biopsy can be safely collected and evaluated if indicated. In galactosemia, there is macrovesicular steatosis associated and unspecific findings of NC, including ductular reaction [9,136,137]. In hereditary fructose intolerance, there is panlobular macrovesicular steatosis associated with portal fibrosis, ductular reaction, lobular fibrosis with regenerative nodules, pseudoacini, necrosis with little inflammation [138], and in tyrosinemia type 1, histopathology evaluation shows macrovesicular steatosis associated with pseudoacini, hemosiderosis and varying degrees of hepatocellular necrosis and apoptosis. Fibrosis develops soon, eventually progressing to micronodular cirrhosis [92,139].

The ductopenic pattern must be adequately defined because, although usually associated with prominent cholestasis, the latter can eventually subside. The prognosis of patients with the syndromic form of bile duct paucity is affected, additionally to the liver disorder, by the complications of the extrahepatic disease manifestations [44]. The characteristic bile duct paucity develops over time, being found in only 60% of livers from 6-month-old infants, but up to 95% of the livers from affected patients beyond this age [140,141].

Later in life, microscopic findings may be heterogeneous with bile duct paucity areas coexisting with other regions exhibiting normal portal spaces or just a tenuous ductular reaction [140,142]. Several other neonatal cholestatic diseases can present paucity of bile ducts and remember that this finding is also observable in normal young infants, especially in premature babies [143,144].

The hepatic storage pattern is an important biopsy target for diagnosis in non-neuropathic storage diseases such as Gaucher and Nieman-Pick type C [35,125] (Supplementary Material, Table S3), while neurometabolic storage diseases rarely require a liver biopsy because the pathologic evidence is present in more accessible tissues. In storage diseases, there is hepatomegaly attributable to cytoplasmic expansion by accumulated material in different liver cell groups separately or in combinations depending on the etiology. Suggestive findings of storage diseases can be mimicked by other conditions (Table S3). PN-associated neonatal cholestasis can lead to hepatocellular lipid and lipofuscin accumulation. Ballooning and pseudoxanthomas of NC can be confused with storage findings. Steatosis and eosinophilic protein in the endoplasmic reticulum must also be distinguished. Even normal conditions may be confounded with storage diseases, such as stellate cells with a foamy appearance in perisinusoidal space due to excessive vitamin A deposition, or the nuclear hyperglycogenation of periportal hepatocytes observable in young infants [125].

**Figure 10:**
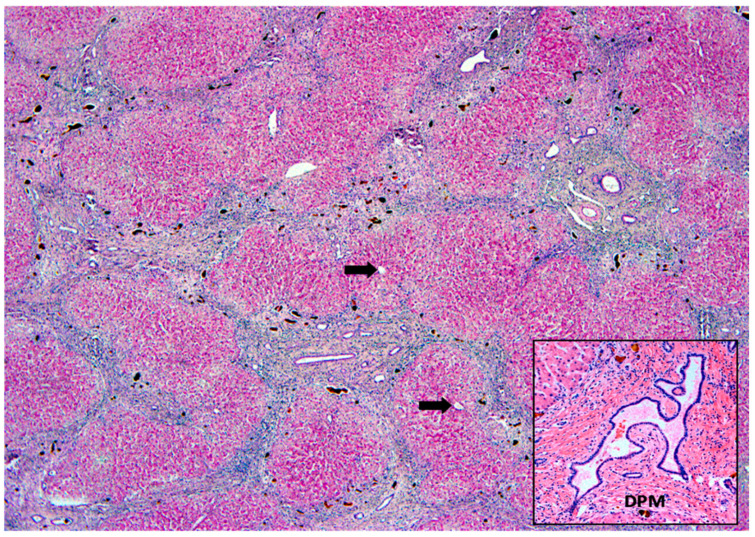
**Liver histopathological picture in Autosomal recessive polycystic kidney disease, with congenital hepatic fibrosis**. Observe the normal architecture within lobules presenting terminal hepatic veins (arrows) and the plugs in biliary structures of fibrous septa. Detail of ductal plate malformation (DPM) at the right lower corner. Masson’s trichrome, lens 10×, and 20×. Obtained at the Department of Pathology, Hospital de Clínicas de Porto Alegre, Brazil, and published under permission of such Service.

**Figure 11:**
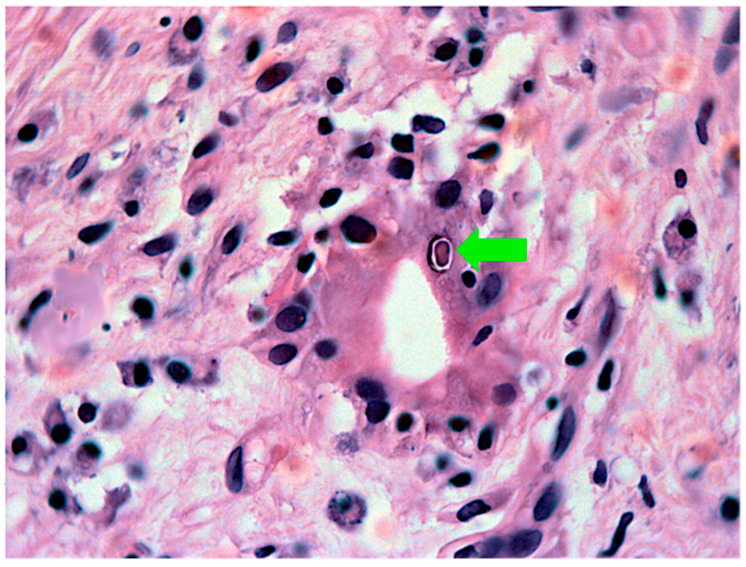
**Viral inclusion** (**Cytomegalovirus, green arrow**) **in a biliary ductule of an infant with neonatal cholestasis.** H-E, lens 100×, using oil immersion. Obtained at the Department of Pathology, Hospital de Clínicas de Porto Alegre, Brazil, and published under permission of such Service.

Additional histopathological patterns indicative of the etiology of NC, including the presence of ductal plate malformation and viral inclusions, are shown in Figure 10 and Figure 11, respectively.

## 5. Conclusions

The differential diagnosis of neonatal cholestasis includes a wide variety of entities and different steps. In all the steps, there is the need to integrate clinical-laboratory tests, including ordinary biochemical and complex enzymatic methods, as well as image and histopathological studies. From the 1990s, genetic tests became increasingly available and cheaper, helping the pathophysiological understanding of neonatal cholestatic disorders and the deciphering of distinct phenotypes previously unrecognized.

One consequence of using genetic tests is that we are uncovering human genetic complexities, for instance, the fact that the presence of a pathogenic variant in a set of patients does not imply a common phenotype between them. A genetic disease can present diverse clinical presentations. Still, a reasonably large group of PFIC cases currently elude genetic diagnosis, maybe due to unknown pathogenic variants or to others presently not implicated with PFIC. Thus, there is a constant learning curve about the methodology to investigate patients with neonatal cholestasis. In addition, this is true not only for NGS but for all diagnostic tests, concerning the deciphering of pathophysiological details. No diagnostic test is 100% accurate in every step of the differential diagnosis of neonatal cholestasis, and the adequate integration of diverse diagnostic methods presently is the correct approach, even the histopathological study of a safe percutaneous liver biopsy. Crucial is to be aware of the difficulties and pitfalls related to each of the investigation methods, even those involving the most sophisticated technologies.

## Figures and Tables

**Table 1:** Differential diagnosis of neonatal cholestasis.

Intrahepatic Cholestasis
**1. Idiopathic Neonatal Hepatitis**
**2. Disorders in Embryogenesis of Biliary Structures** Alagille syndrome (JAG1; NOTCH2); Cholestasis lymphedema syndrome (Aagenaes syndrome). **Ciliopathies:** Ductal plate malformations: ARKPD (PKHD1); Caroli (disease and syndrome); Joubert syndrome with Congenital hepatic fibrosis (COACH); Meckel syndrome; Renal-hepatic-pancreatic dysplasia (NPHP3). Neonatal sclerosing cholangitis without ichthyosis (DCDC2).
**3.Disorders in Primary Bile Acids Synthesis and Conjugation** **Synthesis**: 3β-Hydroxy-Δ5-C27-steroid oxidoreductase deficiency (HSD3B7); Oxosteroid 5β-reductase deficiency (AKR1D1); Sterol 27-hydroxylase deficiency – Cerebrotendinous xanthomatosis (CYP27A1); Oxysterol 7α-hydroxylase (CYP7B1) deficiency; 2-Methylacyl-CoA racemase deficiency (AMACR). **Conjugation**: Bile acid-CoA ligase deficiency—Familial hypercholanemia (BAAT and TJP2).
**4. Transport and Secretion of Cholephilic Compounds** **Nuclear receptors regulation**: PFIC5 (functional defect of FXR, gene NR1H4). **Bile salts intracellular traffic**: PFIC6 (myosin VB protein, gene MYO5B); Arthrogryposis, Renal Dysfunction, and Cholestasis (apical polarity maintenance, genes VPS33B, VIPAS39). **Canalicular membrane secretion**: **bile salts**—BSEP protein (PFIC2, BRIC2, gene ABCB11); **phospholipids**—MDR3 protein (gene ABCB4); cholesterol—sitosterolemia (ABCG5, ABCG8).
**5. Hepatocellular Junctional Complexes** PFIC4 (TJP2); NISCH syndrome (CLDN1).
**6. Complex or Multi Organic Disorders** Phosphatidylserine translocation disorder: PFIC1, BRIC1 (ATP8B1); Arthrogryposis, Renal Dysfunction, and Cholestasis (VPS33B, VIPAS39); Cerebrotendinous xanthomatosis (CYP27A1); Congenital Defects of Glycosylation (CDG) (ALG3, ALG8, GLS1, PMM2, MPI, COG1, COG7, ATP6AP1). **Peroxisomal disorders**—Zellweger spectrum disorder/neonatal adrenoleukodystrophy/neonatal Refsum disease/Heimler syndrome (PEX gene family). **Neonatal intrahepatic cholestasis caused by CITRIN deficiency (NICCD)**—Citrullinemia type II (SLC25A13). **Mitochondrial Respiratory chain disorder**—Mitochondrial depletion syndrome (DGK, POLG, MPV17, DGUOK); Respiratory chain complex III deficiency—GRACILE syndrome (BSC1L); Long-chain 3-hydroxy acyl-CoA dehydrogenase (LCHADD) deficiency (HADHA).
**7. Metabolic Liver Diseases** **Involving biliary system**—Alpha 1-antitrypsin disease (SERPINA 1); Cystic fibrosis (CFTR, ∆F508 variant). **Not involving biliary system**—Glycogen storage disease type IV (GBE1). **Metabolic Intoxication**—Galactosemia (GALT); Hereditary Fructose Intolerance (ALDOB); Tyrosinemia 1 (FAH). **Endocrine Disease**—Hypothyroidism; Hypopituitarism. **Lipid metabolism disorder (storage)**—Wolman syndrome (LIPA); Niemann-Pick disease (NPC 1 and 2); Gaucher disease type II (GBA); Farber disease type IV (ASAH1).
**8. Environmental: Congenital Infections** **Bacterial**—Syphilis; bacterial sepsis; urinary tract infection; Tuberculosis; Listeriosis. **Viral**—Cytomegalovirus; Rubella; Herpes Simplex; Hepatitis (A, B, C); HIV; Parvovirus B19; Varicella zoster; Paramyxovirus; Enteric viral sepsis; Echovirus; Coxsackievirus; Adenovirus. **Parasitic**—Toxoplasmosis.
**9. Immune Disorders** Neonatal Lupus erythematosus; Neonatal Hepatitis with Autoimmune hemolytic anemia; Gestational alloimmune liver disease (GALD).
**10. Others** Transient cholestasis. Parenteral nutrition-associated liver disease (PNALD). Liver cirrhosis; Histiocytosis X; Fibrosing Hepatitis with Transient Leukemia (Trisomy 21); Shock and Hypoperfusion; Neonatal asphyxia; Intestinal obstruction; Drug induced-liver injury (DILI).
**Extrahepatic Obstructive Cholestasis** Biliary atresia. Choledochal cyst; Spontaneous perforation of the common bile duct; Biliary sludge/mucous plug; Cholelithiasis.
Source of genetic information: https://panelapp.genomicsengland.co.uk/ Additional References—[21,22]

**Table 2:** The first line of the differential diagnosis between Biliary atresia and intrahepatic neonatal cholestatic disorders.

**(1) Suggestive findings of BA** General appearance: healthy, thriving, eutrophic. Stools: Acholic stools (7-day observation with stool color card). Hepatobiliary ultrasound: triangular chord (triangular or tubular, not vascularized, echogenic density at the porta hepatis), abnormal/absent gallbladder, hypertrophied hepatic arterial lumen in the hilum, subcapsular blood flow. Invasive procedures: percutaneous liver biopsy (histopathologic obstructive pattern). Novel approaches: ◦ ELISA: Matrix Metalloproteinase 7 (MMP-7) ◦ Abdominal Laparoscopy: subcapsular spider telangiectasia with dilated arterioles. **Exclusion of**: ◦ Gestational intercurrences: maternal cholestasis, acute fatty liver, or infection. ◦ Neonatal data: low APGAR score, clinical intercurrences (low birth weight, sepsis, intestinal anomalies or failure, impossible enteral feeding, use of parenteral nutrition). ◦ Drug-induced liver injury or Herbal-induced liver injury. ◦ Systemic signs of acute disease. ◦ Decreased or normal GGT. ◦ Normal colored stools. **(2) First-line tests for differential diagnosis of NC** **Newborn screening (NBS) results** **Blood tests** Aminotransferases, GGT, Alkaline phosphatase, albumin, glucose, ammonia. Blood count, platelet count, reticulocytes, Coombs test. Coagulation: PT/INR, aPTT, fibrinogen, antithrombin III. Blood electrolytes, calcium, phosphate, magnesium. Lactate, pyruvate, ketones, urea, uric acid, creatinine, ferritin, iron level, transferrin saturation, creatine kinase, lipase, alpha-fetoprotein. Pulse oximetry, blood gases. Protein electrophoresis. **Urine** Urinalysis. Reducing substances in urine (baby under normal diet). Serology (IgG/IgM) HBV, HCV, EBV, CMV, HSV types 1/2, Parvovirus B19, Rubella, HIV, Toxoplasma gondii. **Nucleic acids**: EBV-DNA, CMV-DNA, HSV types 1/2 DNA. **Cultures** (blood, urine, cutaneous and mucosal lesions). **Ultrasound** (abdominal, hepatobiliary, echocardiogram). **Others** ◦ GALT enzyme activity and galactose-1-phosphate levels in erythrocytes, blood galactose. ◦ Serum A1AT levels, protease inhibitor (PI) typing through polyacrylamide isoelectric focusing. ◦ Sweat electrolytes, fecal elastase, immunoreactive trypsinogen. ◦ Ophthalmologic examination with fundoscopy. ◦ Thorax-Rx, Skull-Rx. ◦ Invasive procedures. **Observation**—Early blood sample for DNA extraction must be early collected for expanded sequencing of suspected diseases (A1ATd, Galactosemia, cystic fibrosis, etc.).
References: [7,24,25,26,27]

## Data Availability

Not applicable.

## Supplementary Materials

The following supporting information can be downloaded at: https://www.mdpi.com/article/10.3390/healthcare10102012/s1, Table S1: Clinical-laboratory data suggestive of specific diagnostic diseases or groups of diseases, Table S2: Histopathological findings associated with intrahepatic neonatal cholestasis, Table S3: Histopathologic liver findings at the presentation of some storage diseases causing NC. Figure S1. Bile acid synthesis, metabolism and bile flow, showing the genes associated with the different physiological steps. Abbreviations: (Images) ABC-ATP Binding Cassette (Subfamilies B, C, G); AE2-anion exchange protein 2; ASBT-Apical Sodium Dependent Bile Acid Transporter; BSEP- bile salt export pump; ATP8B1-ATPase Phospholipid Transporting 8B1 (or FIC1-familial intrahepatic cholestasis 1-associated protein); BA-bile acid; CFTR-Cystic Fibrosis Transmembrane Conductance Regulator; ER-endoplasmic reticulum; FGF19-fibroblast growth factor 19; MDR-Multidrug-resistant protein; MRP-multidrug resistance-associated protein; NTCP-Na+-taurocholate co-transporting polypeptide; OATP-organic anion transporting peptide; OST α/β- organic solute transporter alpha and beta; PVP-peribiliary vascular plexus; T/G-taurine oy glycine. (Gene boxes 1–3)–see https://www.genecards.org/ and https://panelapp.genomicsengland.co.uk/. [21,22].
