# Supplementary material for: Protocols of Investigation of Neonatal Cholestasis—A Critical Appraisal
Source: Healthcare (Basel). 2022 Oct 12;10(10):2012. doi: 10.3390/healthcare10102012 (PMC9602084; doi:10.3390/healthcare10102012)
Supplement: Supplementary file 1 [file healthcare-10-02012-s001.zip › healthcare-1913793-supplementary.pdf]

Supplementary Material

TABLES

Table S1. Clinical-laboratory data suggestive of specific diagnostic diseases or groups of diseases

S1.a ACUTELLY ILL

Infection (suspecting of herpesvirus: treat empirically);

Neonatal hemochromatosis (most cases Gestational Alloimmune Liver Disease)

Hemophagocytic lymphohistiocytosis

- **Healthy interval:** Galactosemia, Hereditary Fructose Intolerance, tyrosinemia type 1, disorders of amino acid metabolism, organic aciduria, urea cycle defects
- **Present at birth / first day of life**
  - Reduced fasting tolerance: fatty acids oxidation defects, disorders of ketogenesis
  - Disturbed metabolism of complex molecules: Lysosomal storage disease, Peroxisomal disorders, Congenital disorders of glycosylation
  - Disturbed energy metabolism:
    - Mitochondrial disorders:
      - respiratory chain disorders;
      - urea cycle disorders (OTC deficiency, citrin deficiency);
      - fatty acid oxidation defects (MCAD deficiency, LCHAD deficiency, Ornithine carbamoyltransferase deficiency) - neurological/systemic involvement with lactic acidosis.

Cholestasis + Acute liver failure - ↑↑↑ BD, fast ↑ of aminotransferases, coagulopathy (INR ≥ 1.5)* unresponsive to vitamin K. In babies older than 28 days: encephalopathy may be identified.

- or INR ≥ 2, even without encephalopathy;
- In addition, hypoglycemia and ↑ serum ammonia levels.

**S1.b CLINICAL SIGNS SUGGESTIVE OF SPECIFIC CAUSES OF NEONATAL CHOLESTASIS**
**Hypoglycemia**<br><br>Infection: acute liver failure; Galactosemia; Tyrosinemia type 1; Hereditary fructose intolerance; disorders of amino acid metabolism (including Tyrosinemia type 1); mitochondrial disorders; Congenital disorder of glycosylation 1B; Panhypopituitarism.<br><br>● With ↓ ketone bodies- fatty acid oxidation defects; mitochondrial depletion syndrome.
**Sepsis-** cholestasis secondary to sepsis; Gram- (E. coli), recurrent or prolonged: Galactosemia.
**Asphyxia of an unknown cause or sudden respiratory distress immediately after birth –** mitochondrial disease
**Diarrhea**<br><br>**Acute-** infection; PFIC1; Cystic fibrosis; Galactosemia; Tyrosinemia type 1; acute liver failure.<br><br>**Chronic with steatorrhea-** Bile acid synthesis defects; congenital defects of glycosylation.<br><br>**Intractable diarrhea and cholestasis**- MYO5B (mixed phenotype).
**Vomiting**<br><br>Galactosemia; Tyrosinemia type 1; Congenital disorder of glycosylation 1b; acute liver failure.
**Gastrointestinal hemorrhage at presentation of neonatal cholestasis**<br><br>Tyrosinemia type 1, Primary bile acid synthesis defect (type 1), acute liver failure.

**High-grade hepatomegaly**<br><br>Primary bile acid synthesis defects; Tyrosinemia type 1.<br><br>**Storage liver diseases-** Congenital disorders of glycosylation; Glycogen storage disorders; Bile acid synthesis defects; Wolman syndrome; Niemann-Pick disease (NPC); Gaucher disease type II, Farber disease type IV.<br><br>**Associated with splenomegaly:** Hemophagocytic lymphohistiocytosis; congenital dyserythropoietic anemia type 1.
**S1.c DYSMORPHISM**<br><br>**Chromosomal disorders**- trisomies (18 and 21);<br><br>**Genetic syndromes-** Alagille, ARC, Aagenaes, Smith-Lemli-Opitz;<br><br>**Congenital disorders of glycosylation;**<br><br>**Lysosomal storage disorders;**<br><br>**Mitochondrial disorders** (not in Gracile syndrome);<br><br>**Peroxisomal disorders** – Zellweger spectrum disorders;<br><br>**Organic acidemia** - Glutaric acidemia type II.
**S1.d CUTANEOUS MANIFESTATION**<br><br>**NISCH syndrome -** ichthyosis, alopecia and hypotrichosis.<br><br>**Hemophagocytic lymphohistiocytosis-** erythroderma, purpuric maculae, morbilliform eruptions.
**S1.e DISTINCT ODORS**<br><br>Maple syrup urine disease (maple syrup); Isovaleric acidemia or glutaric acidemia type II (Sweaty feet); Tyrosinemia type I (rancid butter).

**S1.f NEUROLOGICAL SIGNS (convulsions, vomiting, weak cry, recurrent apnea, irritability, lethargy, hypotonia) -** Infection; post-asphyxia; Peroxisomal disorders; Mitochondrial disorders; Lysosomal storage diseases
**S1.g LABORATORY RESULTS SUGGESTIVE OF SPECIFIC CAUSES OF NEONATAL CHOLESTASIS – consider specific TGS**
**<u>Blood</u>**
• **GGT serum levels, bile acids levels (see Targeted Genetic Testing below)**<br>↓ **serum cholesterol-** Smith-Lemli Opitz syndrome, PFIC 1 and PFIC 2, peroxisomal disorders, acute liver failure<br><br>↑↑↑ **ammonia:** urea cycle disorders.<br><br>↑ **Creatine kinase:** mitochondrial disorders; fatty acids oxidation defects.<br><br>↑ **α -fetoprotein:** tyrosinemia type 1; gestational alloimmune liver disease (100,000–600,000 ng/mL); citrin deficiency.<br><br>↑ **uric acid:** fatty acids oxidation defects; mitochondrial disorders.<br><br>↑ **ferritin:** gestational alloimmune liver disease (↑↑↑); mitochondrial disorders; Haemophagocytic lymphohistiocytosis.<br><br>↓ **transferrin:** Congenital defect of glycosylation 1b; gestational alloimmune liver disease; mitochondrial disorders.<br><br>↑ **carbohydrate-deficient transferrin (CDT, by isoelectric focusing):** Galactosemia; Hereditary fructose intolerance.<br><br>↑ **plasma lysosomal enzymes (aspartylglucosaminidase):** Galactosemia; Hereditary fructose intolerance; Congenital disorders of glycosylation.<br><br>↑ **citrulline –** citrin deficiency.<br><br>↑ **lactate:** mitochondrial disorders (↑ lactate/pyruvate); fatty acid oxidation defects.

↑ **copper:** peroxisomal disorders.<br><br>↓ **sodium,** ↑ **potassium:** adrenal insufficiency (panhypopituitarism).<br><br>↓α**-2-globulin (blood proteins):** A1ATd.<br><br>**cytopenia:** Gaucher disease; Niemann-Pick type C; Haemophagocytic lymphohistiocytosis
<u>**Urine**</u>
**Fanconi syndrome** (↑ glucosuria despite normal serum levels, phosphate, amino acids): tyrosinemia type 1, hereditary fructose intolerance, mitochondrial disorders, drugs.
**S1.h TARGETED GENETIC TESTING (with classification based on laboratory tests) – From references 76, 77.**
**S1.h.1 Normal or ↓ GGT**
<u>**With ↑ bile salts (plasma)**</u><br><br>• PFIC 1, BRIC1 - Protein: FIC1; Gene: ATP8B1<br>• PFIC 2, BRIC2 - Protein: BSEP; Gene- ABCB11<br>• TJP2 - Protein: TJP2; Gene- TJP2<br>• NR1H4- – Protein: FXR; Gene: NR1H4<br>• Myosin VB **–** Protein: Myosin VB; Gene: MYO5B<br>• Transaldolase deficiency (TALDOD) **–** Protein: TALDO; Gene: TALDO1<br>• Familial hypercholanemia, conjugation defect (BAAT)- Protein: BAAT; Gene: BAAT
<u>**Without increased bile salts (plasma)**</u><br><br>**Defects of Primary bile acid synthesis:**

IMPORTANT: Is indicated a confirmatory assessment of bile acid intermediates and anomalous bile acids through mass spectrometry in urine.

- 3β-Hydroxy-Δ5-C27-steroid oxidoreductase deficiency (HSD3B7);
- Oxosteroid 5β-reductase deficiency (AKR1D1);
- Sterol 27-hydroxylase deficiency;
- Cerebrotendinous xanthomatosis (CYP27A1);
- Oxysterol 7α-hydroxylase (CYP7B1) deficiency;
- 2-Methylacyl-CoA racemase deficiency (AMACR).

S1.h.2 Neonatal cholestasis, with ↑ GGT serum levels, BA-excluded

- Alagille syndrome (JAG1; NOTCH2)
- A1ATd (SERPINA 1)
- PFIC3 (ABCB4)
- Cystic fibrosis (CFTR)
- NISCH syndrome (CLDN1)
- Isolated neonatal sclerosing cholangitis (DCDC2)
- Citrin deficit (SLC25A13)
- ARC (VPS33B; VIPAR)
- Transaldolase deficiency (TALDO1)
- Niemann-Pick C (NPC1, NPC2)
- Gaucher disease (GBA)
- Wolman syndrome (LIPA)
- Farber disease (ASAH1)
- Glycogen storage disease IV (GBE1)
- Galactosemia (GALT),
- Hereditary fructose intolerance (ALDOB),
- Tyrosinemia type 1 (FAH).
- MCAD deficiency (ACADM)
- Sitosterolemia (ABCG5)

• Urea cycle defects (neonatal)- Citrin deficiency (SLC25A13)<br>• Mitochondrial Complex III deficiency (BCS1L)<br>• Renal-hepatic-pancreatic dysplasia syndrome (Ivemark's syndrome) (NPHP3)<br>• Zellweger spectrum disorder (PEX genes)<br>• Congenital defects of glycosylation (ATP6AP1, CCDC115, SLC37A4, MPI, PMM2, PGM1, ALG1, ALG3, ALG6, ALG8, ALG13, ATP6AP2, COG1, COG4 COG5, COG6, COG7)<br>• <u>WES is indicated in uncertain/complex diagnoses, maybe heredity-dependent without a specific diagnosis available. In this case, integration between clinical e laboratory geneticist teams, and trio analyses are recommended.</u>
**References- 35, 97, 98, 110 - 118**

Table S2. Histopathological findings associated with intrahepatic neonatal cholestasis

<u>Unspecific Hepatitic (inflammatory) pattern in NC</u> - Bile pigments in hepatocytes and Kupffer cells, and canalicular bile plugs; giant cell transformation, pseudoacini, feathery and ballooning hepatocytic degeneration and biliary infarcts:

Idiopathic neonatal cholestasis, Biliary atresia, A1ATd, the initial presentation of metabolic disorders (Niemann-Pick disease type C, Zellweger spectrum disorders, PFIC 2, bile acid synthetic disorders (BASD), neonatal hemochromatosis, cystic fibrosis, ARC syndrome, citrin deficiency, OTC deficiency, and tyrosinemia) and adrenocorticotropic hormone (ACTH) deficiency.

<u>Findings suggestive of specific diseases or groups of diseases</u>

<u>Steatotic pattern</u>

- **Macrovesicular (or mixed) (Fig. 9):** Galactosemia, parenteral nutrition-associated cholestasis, drug-induced cholestasis, A1ATd, cystic fibrosis, Wolman's disease, mitochondrial respiratory chain disorders, fatty acid oxidation disorders, pyruvate-dehydrogenase deficiency, pyruvate-carboxylase deficiency, adrenoleukodystrophy, Refsum disease, organic acidurias, urea cycle disorders, citrin deficiency
- **Microvesicular (Fig 10):** mitochondrial respiratory chain disorder, fatty acid oxidation defects, pyruvate-dehydrogenase deficiency, pyruvate-carboxylase deficiency, organic acidurias, urea cycle defects, valproate toxicity, Reye syndrome.

<u>Bile duct paucity</u>

Alagille syndrome, prematurity, infections (CMV, German measles, syphilis, hepatitis B), metabolic diseases (alpha-1-antitrypsin deficiency, cystic fibrosis, Zellweger syndrome, PFIC, Ivemark syndrome, Prune-Belly syndrome,

hypopituitarism), chromosomal disorders, idiopathic neonatal hepatitis, drug-induced cholestasis.

Hepatocellular necrosis: acute viral hepatitis, metabolic diseases (Hereditary Fructose Intolerance, Galactosemia, Tyrosinemia type 1, fatty acid oxidation defects, mitochondrial respiratory chain disorders).

Nuclear hyper glucuronidation: glycogen storage diseases and urea cycle disorders.

Ductal-plate malformation (Fig. 10): autosomal recessive renal polycystic diseases.

Viral inclusions (Fig. 11): congenital infections.

Table S3. Histopathologic liver findings at the presentation of some storage diseases causing NC

**Disease**	**Histopathological Findings**
A1ATd (PiZ)	Storage of PAS-positive diastase-resistant cytoplasmic globules in periportal hepatocytes (after 12 weeks of life)
Gaucher disease	Enlarged KC and macrophages in sinusoids and portal spaces showing eccentric nucleus and eosinophilic and striated cytoplasm (results from sphingolipid accumulation). Hepatocytes are not involved. Portal and lobular fibrosis.
Wolman disease	Vacuolization of KC and hepatocytes. Foamy histiocytes are situated in portal and periportal areas. Portal and periportal fibrosis, early cirrhosis. In WD: ductular reaction.
Niemann-Pick C	Hepatitic pattern (table S3) with rare foamy macrophages or Kupffer cells (Niemann-Pick cells).
GSD IV	Fibrosis, early cirrhosis. Large, pale, eosinophilic cytoplasmic inclusions surrounded by a halo in most hepatocytes (> periportal zone). Inclusions resistant to diastase may stain with Lugol's iodine.

Based on reference 128. **Abbreviations:** GSD-Glycogen storage disease.

FIGURE

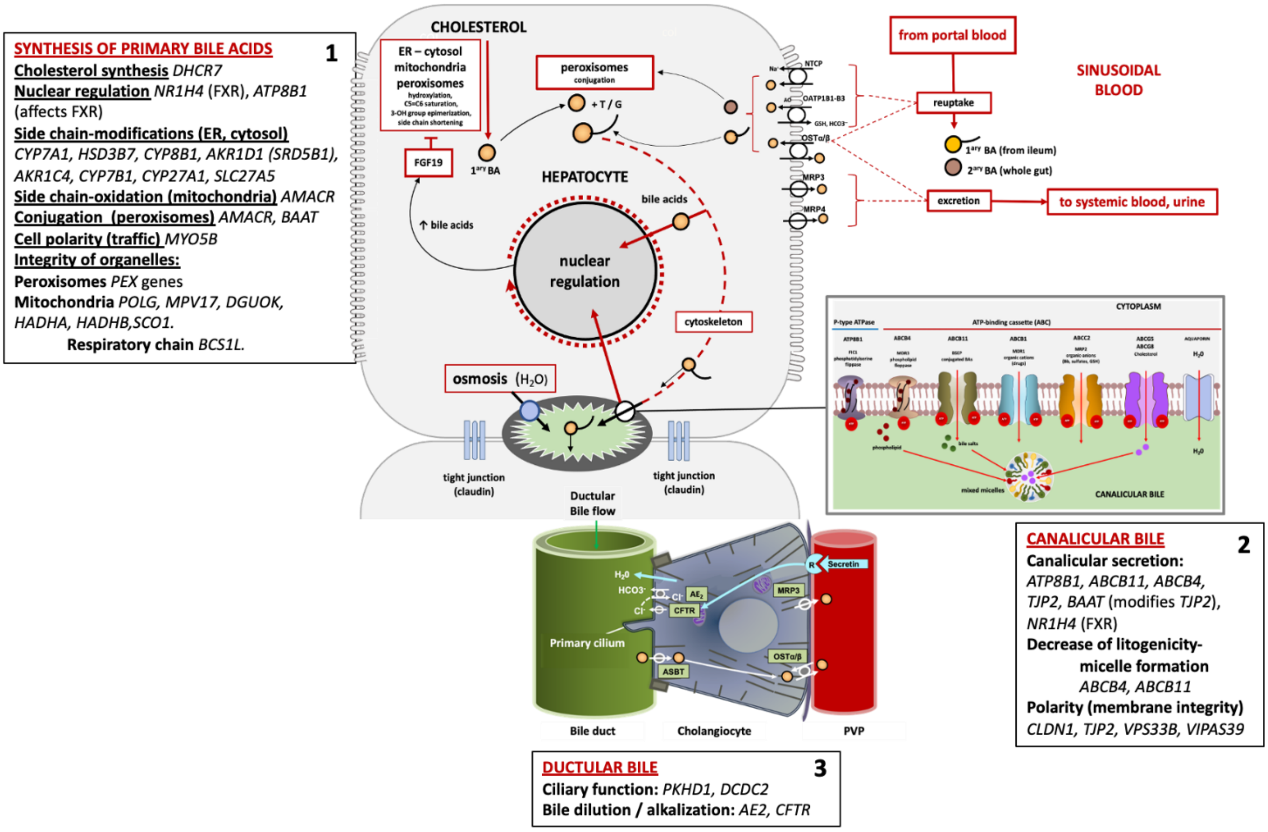

Figure S1. Bile acid synthesis, metabolism and bile flow, showing the genes associated with the different physiological steps. Abbreviations: (Images) ABC- ATP Binding Cassette (Subfamilies B, C, G); AE2- anion exchange protein 2; ASBT- Apical Sodium Dependent Bile Acid Transporter; BSEP- bile salt export pump; ATP8B1-ATPase Phospholipid Transporting 8B1 (or FIC1- familial intrahepatic cholestasis 1-associated protein); BA- bile acid; CFTR- Cystic Fibrosis Transmembrane Conductance Regulator; ER- endoplasmic reticulum; FGF19- fibroblast growth factor 19; MDR- Multidrug-resistant protein; MRP- multidrug resistance-associated protein; NTCP- Na+ -taurocholate co-transporting polypeptide; OATP- organic anion transporting peptide; OST α/β- organic solute transporter alpha and beta; PVP- peribiliary vascular plexus; T/G- taurine oy glycine. (Gene boxes 1-3) – see https://www.genecards.org/ and https://panelapp.genomicsengland.co.uk/. References: 21, 22.
